# Lack of quantitative training among early-career ecologists: a survey of the problem and potential solutions

**DOI:** 10.7717/peerj.285

**Published:** 2014-03-04

**Authors:** Frédéric Barraquand, Thomas H.G. Ezard, Peter S. Jørgensen, Naupaka Zimmerman, Scott Chamberlain, Roberto Salguero-Gómez, Timothy J. Curran, Timothée Poisot

**Affiliations:** 1 Department of Arctic and Marine Biology, University of Tromsø, Tromsø, Norway; 2 Centre for Biological Sciences, University of Southampton, Southampton, United Kingdom; 3 Center for Macroecology, Evolution and Climate, University of Copenhagen, Copenhagen, Denmark; 4 Department of Biology, Stanford University, Stanford, USA; 5 Biology Department, Simon Fraser University, Burnaby, BC, Canada; 6 Max Planck Institute for Demographic Research, Evolutionary Biodemography Laboratory, Rostock, Germany; 7 School of Biological Sciences, Centre for Biodiversity and Conservation Science, University of Queensland, Brisbane, Australia; 8 Department of Ecology, Lincoln University, Canterbury, New Zealand; 9 Département de Biologie, Chimie et Géographie, Université du Québec à Rimouski, Rimouski (QC), Canada; 10 Québec Centre for Biodiversity Sciences, McGill University, Canada

**Keywords:** Education, Statistics, Mathematics, Ecology student, Teaching, University curriculum, Student

## Abstract

Proficiency in mathematics and statistics is essential to modern ecological science, yet few studies have assessed the level of quantitative training received by ecologists. To do so, we conducted an online survey. The 937 respondents were mostly early-career scientists who studied biology as undergraduates. We found a clear self-perceived lack of quantitative training: 75% were not satisfied with their understanding of mathematical models; 75% felt that the level of mathematics was “too low” in their ecology classes; 90% wanted more mathematics classes for ecologists; and 95% more statistics classes. Respondents thought that 30% of classes in ecology-related degrees should be focused on quantitative disciplines, which is likely higher than for most existing programs. The main suggestion to improve quantitative training was to relate theoretical and statistical modeling to applied ecological problems. Improving quantitative training will require dedicated, quantitative classes for ecology-related degrees that contain good mathematical and statistical practice.

## Introduction

Basic tasks in ecological research and management often involve fairly advanced statistics, especially outside of experimental science. Typical examples include capture–recapture models to census populations (Williams, Nichols & Conroy, 2002), or elaborate multivariate statistics to reduce complex datasets of environmental records to a few manageable variables (Legendre & Legendre, 2012). Most papers in mainstream ecological journals today use statistical and computational techniques beyond analysis of variance and simple linear regression. These include, among others: generalized, mixed, or nonlinear regression models; discrete probabilistic models fitted by maximum likelihood; Bayesian statistics and Markov-Chain Monte Carlo [MCMC]; graph-theoretic algorithms for interaction webs; and movement models derived from Brownian motion. We surveyed the July 2012 issues of *Ecology*, *Journal of Animal Ecology,* and *Oikos*, and found these more sophisticated statistical techniques are used in 75%, 95% and 70% of articles, respectively.

Theoretical ecology has been using fairly advanced mathematics since the 1920s and 1930s (e.g., Lotka, 1925; Fisher, 1930; Volterra, 1931), but as a subdiscipline it has, for some time, remained rather separated from the rest of ecological science (Kingsland, 1995). Therefore, the need of theoreticians for mathematics was much greater than that of the average ecologist. In contrast, modern theoretical ecology is more and more connected to ecological data (Hilborn & Mangel, 1997; Bolker, 2005; Codling & Dumbrell, 2012), and this fusion of theoretical and statistical models increases the need for many ecologists to have a detailed understanding of the theoretical and statistical sides of their discipline.

Examples of a tighter link between theory and data abound in population dynamics (e.g., population projection models, Caswell, 2001), behavioral sciences (e.g., hidden Markov models, Patterson et al., 2008), and community ecology (e.g., neutral models, Hubbell, 2001; graph theory for food webs, Dunne, 2006). These fields have a long tradition of the use of quantitative methods, but the rise of improved and often freely available software has made complex mathematical and computational tools accessible to all. The trend is clear from the recent proliferation of textbooks designed to teach students modern ecological modeling and statistics (e.g., Gotelli & Ellison, 2004; Clark, 2007; Otto & Day, 2007; Bolker, 2008; Stevens, 2009; Matthiopoulos, 2011), and the creation of new methodological journals (e.g., *Methods in Ecology and Evolution*). Similarly, the open-source statistical programming language R (R Core Team, 2013) has been embraced by much of the ecological community. Fifty years ago, Pielou (1969) thought that ecology was becoming a “mathematical” subject. While it is unclear whether ecology is truly more mathematical in nature, the requirement for statistical and computational skills in postgraduates has certainly increased, and so did the rate at which new quantitative methods are developed and published (see references above). In the current landscape of ecological research, a lack of mathematical literacy can prohibit access to a large part of the ecological statistics and theoretical literatures, and run the risk of producing analyses that are considered sub-standard by reviewers and editors. Outstanding research can, needless to say, still be performed with limited mathematical background, but there is undeniably an impression that quantitatively intensive ecological research is becoming more dominant. This poses a problem for ecology as a whole: equations remain a barrier to effective communication between empiricists and theoreticians/statisticians (Fawcett & Higginson, 2012), even if these problems are, perhaps, not as strong as when highlighted by ecological pioneers such as Elton (Kingsland, 1995).

Given the trend for more quantitative research in ecology, one might expect current ecology students to receive training rich in mathematics, statistics and programming. By mathematics, we mean both “pure” topics such as calculus, algebra, and probability, and more applied topics usual in theoretical ecology such as dynamical systems. By statistics, we mean techniques used for the collection, organization, and interpretation of data, covering therefore both exploratory (e.g., principal component analysis) and inferential statistical techniques (e.g., the linear model). Programming refers both to algorithms (e.g., the “for loop”) and their practical implementation (e.g., how to use R or Python). With the increase in the availability of advanced methods, quantitative training ought to focus on (i) understanding how these methods work and (ii) when to use them. However, many ecology students at the undergraduate or graduate level do not have the required background to formulate statistical or theoretical models, or even to understand their properties (Ellison & Dennis, 2010). As such, undergraduate courses in ecology can resemble storytelling without strong mathematical or statistical foundation, which is far removed from current ecological science. Based on their experience, Ellison & Dennis (2010) advocate, for students to reach “statistical fluency”, the teaching of ecological statistics only after a two-semester calculus course at undergraduate level, possibly supplemented by linear algebra and probability theory for graduate students. However, data on the level of quantitative training that early career ecologists themselves consider appropriate are rare. Are more undergraduate mathematics classes the answer? How many ecologists are distressed by their lack of formal mathematical and statistical training? Early-career scientists are well equipped to comment on these issues: they are lead authors on many papers, and therefore deal first-hand with many of the technical issues that arise. Many aspects of their formal education and training are fresh in the memories of early career researchers, and these aspects are likely to reflect current trends. Here we attempt to assess the size of the “quantitative gap” in young scientists through an online survey (see Appendix S1) diffused through various list-serves (see below for details). We wanted to know what early-career researchers (mainly PhD candidates and postdocs) think about the mathematical and statistical training they have received, and what (if anything) they think should be done to improve it.

## Survey design, data, and methods

We designed this survey as a short online questionnaire (see Appendix S1). The questionnaire was anonymous and voluntary. No identifying questions needing ethical approval were asked. The guidelines of Norwegian research ethics (country of first author’s institution) were followed. The survey was launched on the 13th of February, 2012, through the INNGE network (http://innge.net). The last answers were recorded on the 10th of April, 2012, with a peak in participation after diffusion on the American ECOLOG-L mailing list (16th of February, 2012). After ECOLOG-L, the survey was forwarded to a number of networks including the Indian YETI mailing list and members of the French Ecological Society as well as being diffused globally through social media (Twitter) and a number of science-related blogs (including that of the ecological journal *Oikos*). The total number of responses was 937, of whom 250 also left free text comments that we categorized (see “Comments of Respondents”). The data have been deposited as Supplemental Information 1.

Key proportions presented in the paper, and differences between those proportions, are accompanied with their 95% asymptotically normal confidence intervals, using a binomial model (more complex CIs, e.g., Agresti–Coull, are available but those used here are sufficient given the large sample size, Agresti, 2007).

## Control questions: survey composition

### Demographics: education, geography and gender

**Figure 1:**
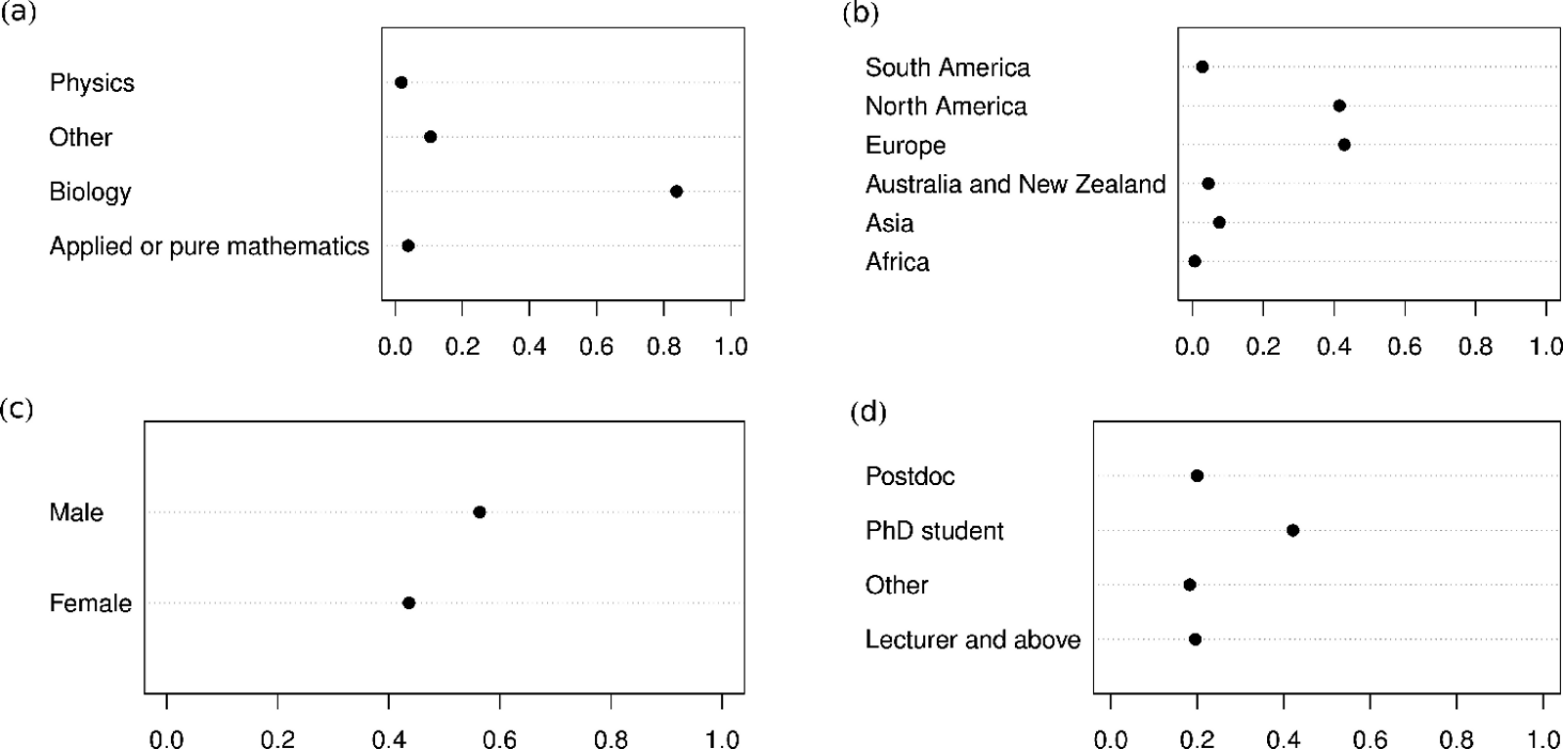
Survey composition. Partitioning of the respondents with respect to (A) background (i.e., discipline of undergraduate studies), (B) geographic origin, (C) gender, and (D) employment status/level.

Most respondents (84%) were trained as biologists (Fig. 1). Nearly half of the respondents are PhD students (42%), with 20% postdocs and 20% lecturers or professors (Fig. 1). Based on free text comments, the category “other” (18%) includes numerous MSc students. The survey contains a relatively balanced provenance according to gender (44% females, CI [40.8;47.2]%). Most respondents are from either Europe or North America (43%: Europe; 41%: North America). There was no general correlation between geography and gender (the results for PhD students suggest only small differences among them in Europe and North America, for example, Fig. S1).

### Involvement in modeling and “mathematics-friendliness”

**Figure 2:**
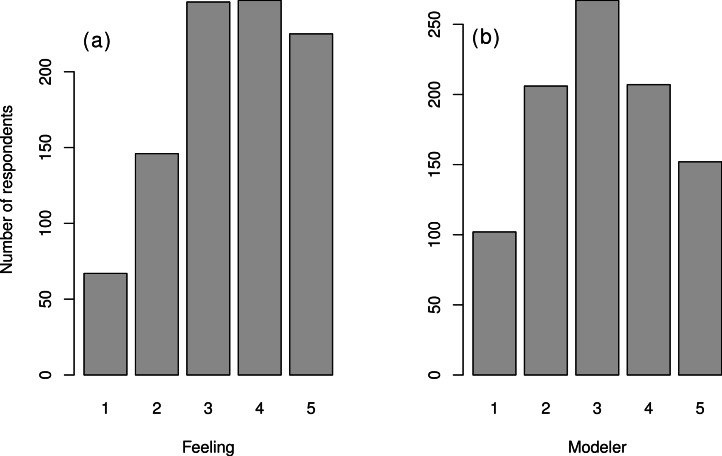
Distribution of variables quantifying attitudes towards mathematics. (A) Distribution of “Feeling” variable (from 1: “really dislike” mathematics to 5: “really like”) and (B) Distribution of “Modeler” variable (1: “do not model” to 5: “specialist modeler”). See Fig. S2 for correlation between these two variables.

A survey such as this could be biased if the respondents predominantly liked or disliked quantitative approaches to ecology. As it was not possible to control the composition of participants with a voluntary survey, we attempted instead to assess the extent of this bias by asking respondents questions about their own feelings about mathematical and statistical training. To do so, we asked the respondents “Rate your feeling towards using equations” and “Rate your involvement in the process of ecological modeling in your field” (Appendix S1 Questionnaire; note that this question also assess statistical models, and not only dynamical ones). The two scores are moderately correlated (Fig. S2, Spearman’s rho = 0.53). We found that most self-identified modelers (Modeler scores 4 and 5) have positive feelings associated with mathematics; conversely, quite a few (42%) of the mathematics-friendly respondents (Feeling score 4 and 5) do not identify as modelers (they have a Modeler score < 4, Fig. 2 and Fig. S2). In passing, we note that more males are modelers or positive towards using equations. Considering only Modeler and Feeling scores 4 and 5, the percentage of females drops to 33% for both variables (this percentage was 44% in the full sample).

## Use of mathematics/statistics and current training

### What are the respondents using mathematics for?

**Figure 3:**
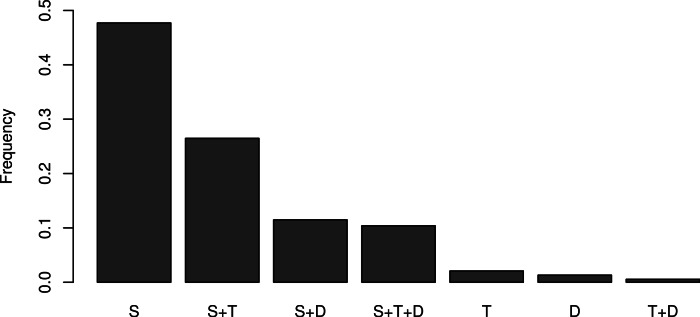
Relative frequency of the uses of mathematics and association between categories. Most respondents use mathematics primarily for statistics (S), and some other for statistics+theory (S+T, 26%), and the remaining 11% for statistics+decision making (S+D) and 10% for statistics+theory+decision making (S+T+D). Pure theoreticians (T) are therefore negligible in the sample.

The first question of the survey reveals that 96% of respondents use mathematics for statistics, 39% use mathematics for theoretical modeling and 24% for decision making overall (see supplementary graphs at https://sites.google.com/site/mathematicsandecologysurvey/summary). A small fraction (11%) use mathematics for decision making but not theoretical modeling (correlations between these variables are shown in Fig. 3). Theoretical work is mostly carried out in combination with other math-intensive practices; very few pure theoreticians responded (2%) and 47% of respondents use mathematics only for statistics (Fig. 3). It is therefore possible that the proportion of theoreticians in our sample is slightly above that of the average population of ecologists, but not overly so.

### Understanding models within one’s field

**Figure 4:**
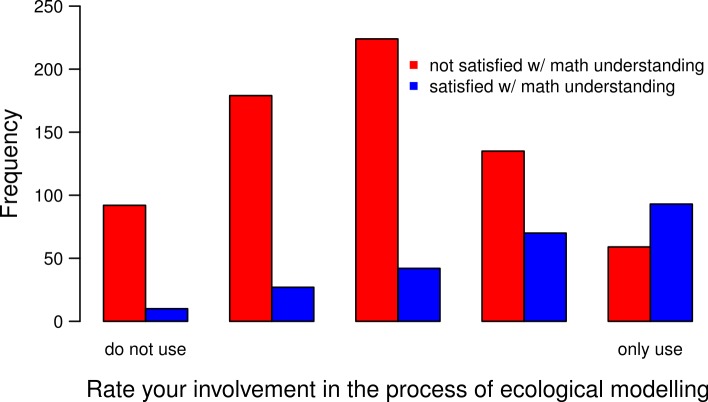
Importance of involvement in modeling on the understanding of mathematical models. The “Modeler” score goes from 1 (“do not use models”, on the left) to 5 (“only use models”, on the right). Red color is associated to dissatisfaction with mathematical understanding and blue to satisfaction.

We asked respondents to assess whether they were satisfied with their understanding of models in their own field; the goal was to assess quantitative understanding in directly relevant areas for them rather than general theory. Based on the response to this question, 75% (CI [73.2;77.8]%) of respondents do not feel satisfied with their understanding of models (and likely with the mathematical training they received). To interpret this number, it is worthwhile to note that humans, including academics, are prone to over-rate their own abilities (van Veelen & Nowak, 2011, and references therein) so, if anything, the 25% of satisfied respondents is an overestimate of true satisfaction with mathematical understanding. Given our large sample size (> 900 participants), these results most likely reflect a true lack of understanding of models within the ecological community. Even among self-diagnosed modeling “specialists” (score 5), only 60% consider themselves satisfied with the mathematical training they received and this figure drops to under 50% for all other “Modeling” groups (Fig. 4). To make sense of this result, consider that 75% of respondents with a mathematics-based undergraduate degree (27 of 36) are, in contrast, satisfied with their understanding of models—though not all of them identify currently as modelers. We found no strong influence of gender (only a 5.6% with 95%CI [−0.045, 0.156] when restricting to Feeling scores 4, 5), and only a weak effect of geography (Fig. S3) on these results. This suggests that such dissatisfaction is international and understanding of mathematical models is strongly dependent on having mathematics classes at the undergraduate level.

### Is there enough mathematics in general ecology courses?

We asked: “In the general ecology courses you have followed, how would you describe the level of mathematics (in retrospect)?” with three possible answers: “Too low”, “Just right”, and “Too high”. We included “in retrospect” because it seems a common experience for ecology students to initially appreciate verbal descriptions of ecological theories and analytical tools, rather than a mathematical description of those same theories using equations. Quite often, students discover later that these concepts and tools involve some fairly advanced mathematics (Ellison & Dennis, 2010). For a number of ecologists, this late discovery seems quite troublesome (see “Comments of Respondents”). Of those surveyed, 75% thought, in retrospect, that the amount of mathematics presented in their ecological coursework was “too low” (22% said “just right” and 2% “too high”). These results do not depend on geographic origin, but are weakly related to whether the participants use mathematics for statistics only or for other purposes as well (7% percent difference, 95%CI: [1%; 13%], Fig. S4).

## What should be done?

### More mathematics and statistics classes

We asked whether there should be more mathematics and statistics in the ecological curriculum. We asked for opinions (“Do you think …”) instead of absolute answers (“Should …”) to allow for more personal inclinations in the responses. The overwhelming majority of respondents want more mathematics courses (91%, CI [89.1;92.9]%) and more statistics courses (95%, CI [93.6;96.4]%). Surprisingly, these percentages (90% for more mathematics and 95% more statistics) do not vary much across categories, and hold for the categories 1 and 2 of the “Feeling” variable (> 200 respondents), who therefore reported disliking the use of equations to construct mathematical models (Feeling = 1: “really dislike”, Feeling = 5: “really like”). More than half of respondents want more mathematics and statistics at both undergraduate and graduate levels (61% for mathematics and 76% for statistics). Additionally, 14% want more mathematics only at the undergraduate level, and another 16% desire more mathematics only at the graduate level. For statistics, 7% want more statistics only at the undergraduate level, and 11% only at the graduate level. In essence, respondents want more mathematical and statistical training. The opinions do not depend much on what people use mathematics for; we found only a 5% difference between respondents using mathematics for statistics-only or other purposes as well (Fig. S4).

### Thirty percent of the ecological curriculum should be mathematics, statistics, or programming

**Figure 5:**
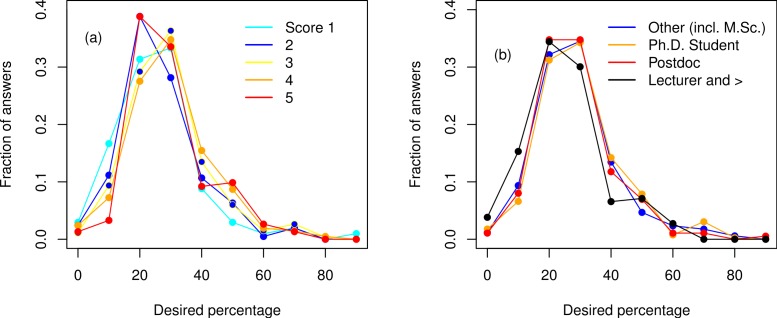
Frequency distribution of the desired percentage of mathematics, statistics and programming (in the ecological curriculum). (A) with respect to involvement in modeling (“Modeler” score, 1: no modeling to 5: specialist), (B) with respect to status/employment level.

To assess what fraction of the university curriculum respondents thought was appropriate to devote to mathematics, statistics, or programming, we asked: “What percentage mathematics, statistics, and programming should approximately cover of the university curriculum of an ecologist, in your opinion?” Given the inherent interdisciplinary nature of ecology, the responses should produce a wide probability distribution whose median indicates the best approximation of a “consensus”. In our results, the median was 30% and the mean 28.3% (two modes at 20% and 30%, Fig. 5). ANOVAs on this fraction, with explanatory factors such “Feeling” or “Modeler”, yielded mostly statistically significant results due to the large sample size, but the magnitude of these effects were very small, nearly all below 4% (for a justification of using ANOVAs given the discrete number of options, see e.g., Norman, 2010). Thus, most respondents, regardless of “Modeller”, “Feeling”, “Status” or “Geographic origin”, agree that one-fourth to one-third of classes in ecology programs should be devoted to quantitative training (Fig. 5).

## Comments of respondents

After carefully evaluating the comments left by 250 out of the 937 respondents, we classified them into four categories (see https://sites.google.com/site/mathematicsandecologysurvey/summary for a selection of emblematic representative comments). Categories 1 and 2 below were pre-determined, as they correspond to alternative teaching strategies (1: Teach mathematics within ecology/highlight ecological relevance of mathematical principles, 2: Increase mathematics requirements/add mathematics classes, as recommended by Ellison & Dennis, 2010). We added categories 3 and 4 to account for other frequently observed comments. Note these categories are not mutually exclusive (below), and some comments (26%) could not be tied to any particular category and were therefore excluded from the following classification.

(1) Teach mathematics for ecologists/biologists (36% of comments). Many respondents feel abstract mathematical/statistical classes, or teachers from pure or applied mathematics, do not bridge the gap between mathematics and application. Some respondents pointed out much of the theory/statistics taught is not particularly applicable to the empirical datasets gathered by ecologists.
(2) Inform “mathematics avoiders” of the quantitative nature of ecology (33% of comments). Many ecology students come to ecology programs hoping to avoid mathematics. Many respondents feel we need to advertise early on to high school and undergraduate students the quantitative nature of ecology-related disciplines. Variant: make classes of mathematics/statistics compulsory.
(3) Teach students how to program (14% of comments). Variant: Use R (R Core Team, 2013), instead of point-and-click statistical packages.
(4) Personal experience in favor of mathematical training (11% of the comments). ‘I wish I had learned more mathematics, I encounter difficulties now’ or ‘I’ve been lucky to learn some mathematics, and that puts me at a huge advantage now.’

The last anonymous comment in the sample speaks for the general sentiment:

“*Given the nature of the field, and despite the outsourcing of modeling to specialists, it is good to at least understand what is going on within the model or behind the model, if not directly programming it yourself. This deeper understanding allows for better theory. It has taken me months of just focusing on statistics/mathematics and models to just get up to speed with fundamentals that I wish had been given during undergrad.*”

## Discussion

Overall, our results indicate that quantitative training in ecology is often insufficient and that arresting this insufficiency requires both extra classes and better integration of quantitative methods within existing programs. Most of our ecological respondents seem to agree with Ellison & Dennis (2010) and Hobbs & Ogle (2011) that calculus is important (and 57% feel they miss notions of calculus). We had expected probability to be the sub-discipline that respondents felt was currently most lacking (see Appendix S1 “Questionnaire”) because ecologists mainly use mathematics for statistics and because probabilistic models are used in both theory and decision-making. Contrary to our expectation, calculus, linear algebra, and even graph theory were also described as areas in need of further training (Fig. S5). One possible explanation for this unexpected result is that ecologists encounter difficulties directly tied to their knowledge in calculus and linear algebra while trying to understand statistics and probability (e.g., partial derivatives and matrices are used in many advanced statistical courses). What is clear, however, is that a few classes sprinkled across disparate modules do not provide the holistic overview of quantitative training as requested by the respondents. Our interpretation of the survey results is in line with the proposed coursework of Ellison & Dennis (2010)—a two-semester course of calculus (broadly defined, including some linear algebra as nearly half our respondents feel a lack in that area) as well as introductory statistics for undergraduates. At graduate level, the proposed additional two-semester sequence of probability and advanced statistics seems very appropriate; but according to our respondents, this course would be better taught with numerous ecological examples. For quantitative training to be successful, our results indicate that we should (1) advertise the quantitative nature of ecology earlier and (2) better connect mathematics and statistics to particular ecological problems and datasets (as suggested in Hobbs & Ogle, 2011). We elaborate on these points below.

Conveying the quantitative nature of ecology to high-school students and undergraduates before they specialize is non-trivial. The comments of our respondents indicate that many aspiring ecologists entered the discipline not only because they loved animal and plant life, but also because they were less inspired by other, more quantitative, physical sciences. We should strive to present more clearly the quantitative nature of the discipline earlier, perhaps as early as high-school (which highlights, in turn, the importance of incorporating more mathematics within ecological courses followed by future teachers). For undergraduate and later graduate students, combining math-intensive activities with fieldwork has also been suggested (Gimenez et al., 2012) as one way of better integrating the quantitative and empirical approaches to ecology and introducing the necessity of both to new students. Moreover, mathematics, statistics and programming are transferable skills that boost employment prospects inside and outside of academia—this cannot be overstressed.

On the practical side, our results indicate that ecologists want mathematics and statistics to be taught by quantitative ecologists so that the curriculum is applied and relevant. This suggests that departments who provide quantitative training via service teaching from mathematicians may not provide the optimal training for their students. We also asked whether programming classes should be taught separately or merged with mathematics and statistics. The results did not show a strong preference (63% merged, 37% separated, with no trend according to respondents’ profiles). Merging classes would allow a clearer integration of programming with practical problems; separated programming classes would promote higher levels of programming ability. One respondent commented: “initially separate, then merged”. This appears to us as a sound proposition, because it allows students not to be overwhelmed at first by simultaneous struggles with computing and statistical/model thinking. As soon as some familiarity with computer programming is established, however, ecology/biology-driven courses help to show students the usefulness of programming (e.g., Valle & Berdanier, 2012) and how the approach can be used to test ecological hypotheses.

Note that we do not imply that basic knowledge in ecology, evolutionary biology, or any related discipline such as geography, physiology or molecular genetics should be replaced in undergraduate curricula by mathematics and statistics. Indeed we do not believe that adding more effective quantitative training precludes the teaching of these fields, and that they would necessarily loose time in favor of quantitative disciplines. Currently, many biological courses require rote learning in e.g., anatomy, morphology, or taxonomy, especially at the undergraduate level. Though memory has to be trained and a background in these biological sub-disciplines is important, the amount of time spent on memorization tasks could likely be reduced. Of course, this holds only true for the majority of undergraduate biology students, some of which will choose ecology at various points in their curriculum; we are certainly not suggesting that veterinarians learn less anatomy. Likewise, taxonomy is very valuable to various fields of biology, and the knowledge of biological diversity should be encouraged: we simply mean that a fraction of the energy applied to remember precisely lists of organisms, organs, tissues, or chemical reactions could be diverted towards learning mathematics, statistics and programming. The fundamentals of these quantitative disciplines are highly transferable to the world of employment in many fields. In some cases, integration with biological courses is possible, see below. One-third of quantitative disciplines seems a good balance for the university curriculum of an ecologist, but specialization can be as late as the master level. Given that biology curriculums make compromises between different specialties, the right fraction of quantitative classes at the undergraduate level, when specialization is late, will likely be found on a case-by-case basis. How best to inferface with physics and chemistry is another open debate (Bialek & Botstein, 2004). However, the needs of other biological disciplines suggest that a more quantitative education in general undergraduate biology is desirable, e.g., neuroscience (Bialek & Botstein, 2004; Hastings et al., 2005) or bioinformatics (Pevzner & Shamir, 2009). It is additionally possible to learn biology while learning math (e.g., biology-inspired calculus, Schreiber, 2009), thus minimizing the time “lost”. Later, students used to a little applied mathematics from population genetics or demography classes naturally become more quantitative, which exemplifies the mutual benefits of combined mathematical and biological training.

## Conclusion

Ecology is moving into an increasingly quantitative era (Hastings et al., 2005), which demands a general review of mathematical, statistical and programming training (Brewer & Smith, 2011). Collaborative research projects and data sets are both expanding in size and complexity, for which we need ecologists trained in state-of-the-art modeling (Hobbs & Ogle, 2011). This survey points to the widespread recognition of the need for better quantitative training in ecology among early-career ecologists, and highlights two useful means to do so: additional mathematics/statistics classes (especially calculus and algebra for undergraduates, when these are absent), and making already existing ecology classes more quantitative, combining mathematical, statistical, and programming concepts with ecological knowledge (see also Anderson et al., 2003, for a more applied perspective). The changing landscape of how data is collected and analyzed in ecology means that ecology departments will need to invest more in the teaching of quantitative methods and concepts. According to our survey, the community would welcome this investment.

## Supplemental Information

10.7717/peerj.285/supp-1 Supplemental Information 1 Main data file Contains the 937 questionnaire answers. The first column provides names for variables.

10.7717/peerj.285/supp-2 Supplemental Information 2 Suggestions of respondents Free comments left by 250 respondents.

10.7717/peerj.285/supp-3 Supplemental Information 3 Classification of respondent suggestions The first column is the suggestions ID in “Suggestion.txt” file, and the 4 remaining columns correspond to the 4 categories referred to in the main text. See legend after comment sign # in the file for more details.

10.7717/peerj.285/supp-4 Appendix S1 Appendix S1 (questionnaire) and Supplementary Fig. S1 to S5
