# Supplementary material for: Lack of quantitative training among early-career ecologists: a survey of the problem and potential solutions
Source: PeerJ. 2014 Mar 4;2:e285. doi: 10.7717/peerj.285 (PMC3961151; doi:10.7717/peerj.285)
Supplement: Appendix S1 [file peerj-02-285-s004.pdf]

Supplementary Information

For Barraquand et al. (2014), Lack of quantitative training among early-career ecologists: a survey of the problem and potential solutions. PeerJ 2:e285; DOI 10.7717/peerj.285. This SI contains the Appendix 1 (questionnaire) and Supplementary Figures S1-5.

Appendix 1

Questionnaire as it appeared online (now closed)

Mathematical training for ecologists

The objective of this survey is to document how ecologists, especially early-career scientists, view their quantitative training, and what are their attitudes towards mathematics. Ecologists often discuss the increasingly quantitative nature of their discipline, and although classes in mathematics and statistics exist for ecologists, there is no clear standard for basic mathematical training. We would like to obtain a better picture of the needs of ecologists, to improve the training in quantitative areas (mathematics, statistics, programming).

Please let us know what you think by filling this short questionnaire!

What are you using mathematics for?
Please check several boxes if you use mathematical modelling in various areas. Do not check any box if you are not using mathematical models of any kind

- ☐ Statistics
- ☐ Theory
- ☐ Decision making

What is your background? As an undergraduate you were studying

- ○ Biology
- ○ Physics
- ○ Applied or pure mathematics
- ○ Other

Rate your feeling towards using equations
To construct a mathematical, statistical, or computational model

	1	2	3	4	5	
you really dislike it	○	○	○	○	○	you really like it

Rate your involvement in the process of ecological modelling in your field

	1	2	3	4	5	
You do not use models	○	○	○	○	○	Modelling is your specialty

Are you satisfied with your understanding of the mathematics behind the models used in your field?

- ○ Yes
- ○ No

In the general ecology courses you have followed, how would you describe the level of

mathematics (in retrospect)?

- ◯ Too low
- ◯ Just right
- ◯ Too high

Do you think more mathematics classes (statistics not included) during the ecological curriculum would be good?

- ◯ No
- ◯ Yes, at undergraduate level
- ◯ Yes, at graduate level
- ◯ Yes, at both levels

Do you think more classes teaching statistics during the ecological curriculum would be good?

- ◯ No
- ◯ Yes, at undergraduate level
- ◯ Yes, at graduate level
- ◯ Yes, at both levels

Should classes in statistics and mathematics be merged with or separated from classes in programming?

- ◯ Merged
- ◯ Separated

What percentage mathematics, statistics, and programming should approximately cover of the university curriculum of an ecologist, in your opinion?

(expressed in %)

0

If you are lacking knowledge in one or severals areas of mathematics that would be useful to you as an ecologist , please indicate which

Categories are Probability (e.g. underlying mathematics behind statistics, stochastic models of any kind), Calculus (e.g. derivatives, integrals), Linear algebra (e.g. matrices), Graph theory (i.e. mathematics of networks), Geometry (e.g. projections for maps, transformation of coordinates), Other (e.g. arithmetics). You can check several boxes. For instance, dynamical systems such as Lotka-Volterra differential equations make use of both calculus and algebra. Please use "Other" only if no other category corresponds.

- ☐ Probability
- ☐ Calculus
- ☐ Linear algebra

- [ ] Graph theory
- [ ] Geometry
- [ ] Other

A little more on yourself. You are currently
The survey is strictly anonymous

- ( ) PhD student
- ( ) Postdoc
- ( ) Lecturer and above
- ( ) Other

You are

- ( ) Male
- ( ) Female

You completed mainly your studies in
(the Europe category includes countries outside EU and includes Russia)

- ( ) Asia
- ( ) Africa
- ( ) Australia and New Zealand
- ( ) Europe
- ( ) South America
- ( ) North America

Any suggestion on mathematical training for ecologists?

Submit

Powered by Google Docs

Report Abuse - Terms of Service - Additional Terms

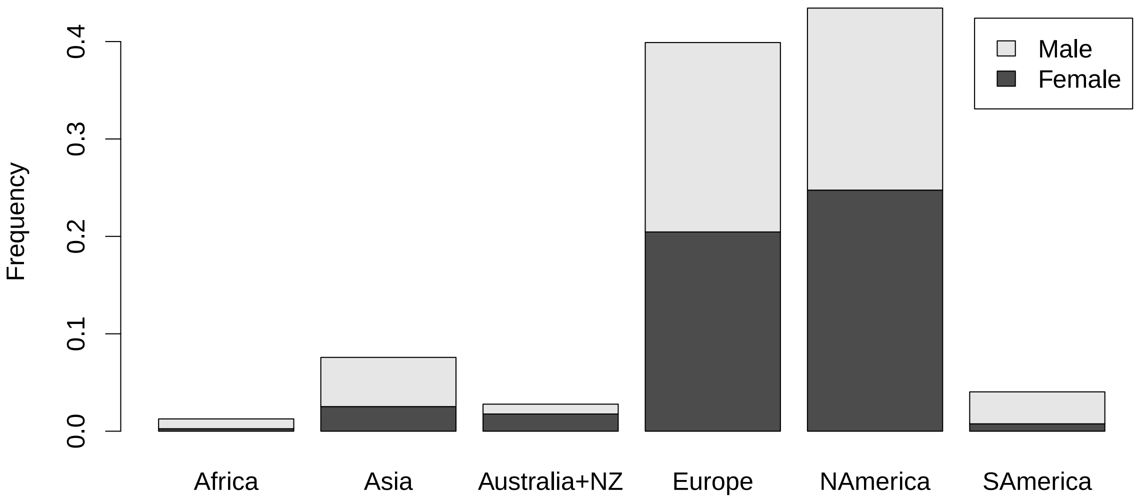

Figure S1: Repartition of sex vs. geographic origin among PhD students (the largest "Status" category), with a bit more females than males in North America (57% females in North America for 51% in Europe, though the 6% difference in proportions is not strongly statistically significant, P=0.3). Other categories such as Postdoc and Lecturer/Professor seem to always show more males than females.

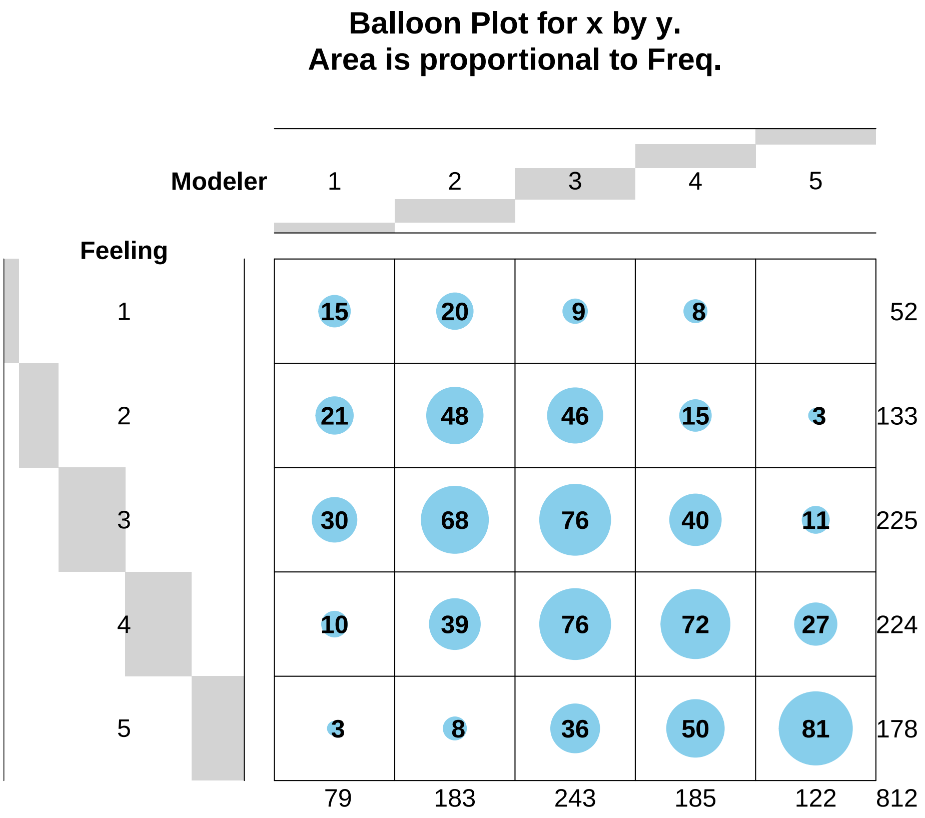

Figure S2: Correlation between involvement in modelling and "mathematics-friendliness". "Modeler" score, answer to question "Rate your feeling towards using equations"; "Feeling" = answer to question "Rate your involvement in the process of ecological modelling in your field".

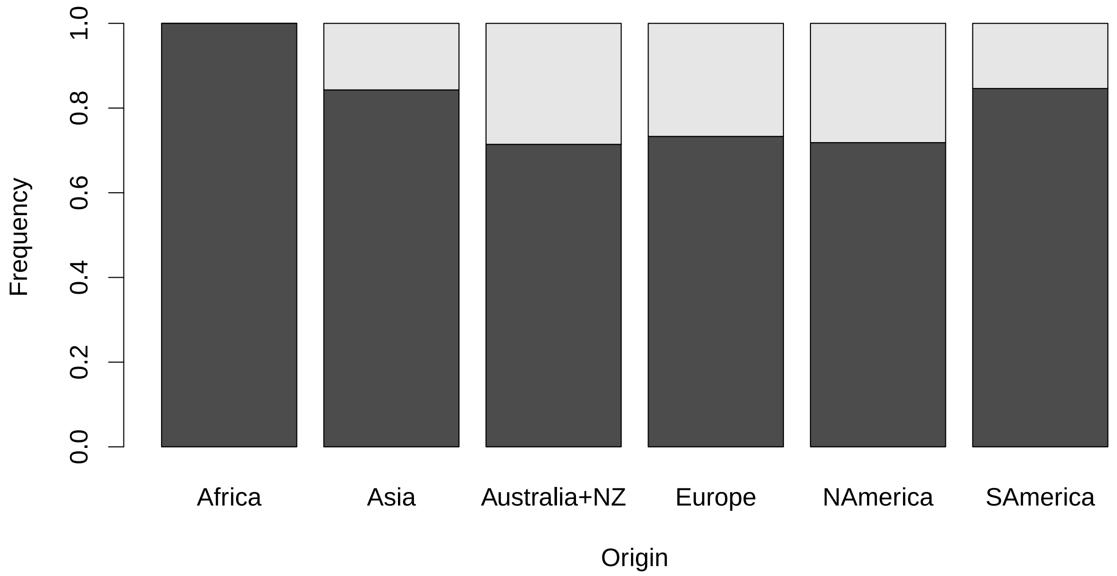

Figure S3: Influence of geographic origin on satisfaction with mathematical understanding of models, normalised bar plot (all counts have been divided by the number of respondents in each geographic group). Dark grey: not satisfied, Light grey: satisfied. Note that Africa, South America, and to a lesser extent Asia have much smaller sample sizes.

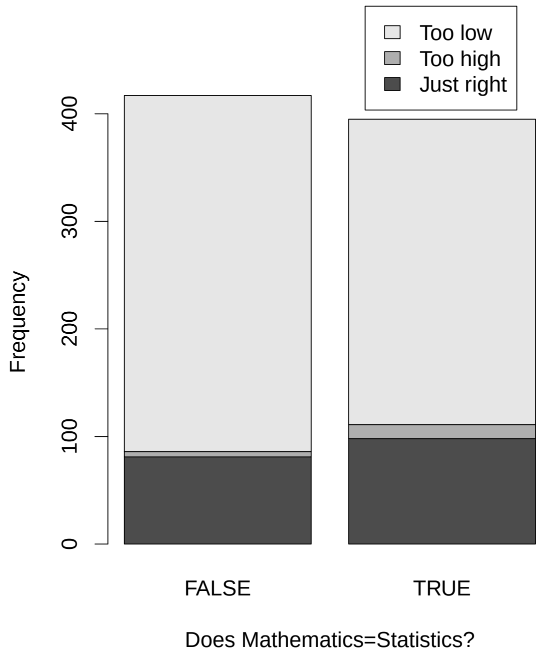

Figure S4: Repartition of opinion on whether the mathematical level is adequate in general ecology courses, as a function of use of mathematics only for statistics, or for other purposes as well (=theory, decision making). A small difference is present, i.e. 79 % respond "Too low " when using mathematics for other purposes as well, and 72 % "Too low" when mathematics are used for statistics only ("TRUE" column).

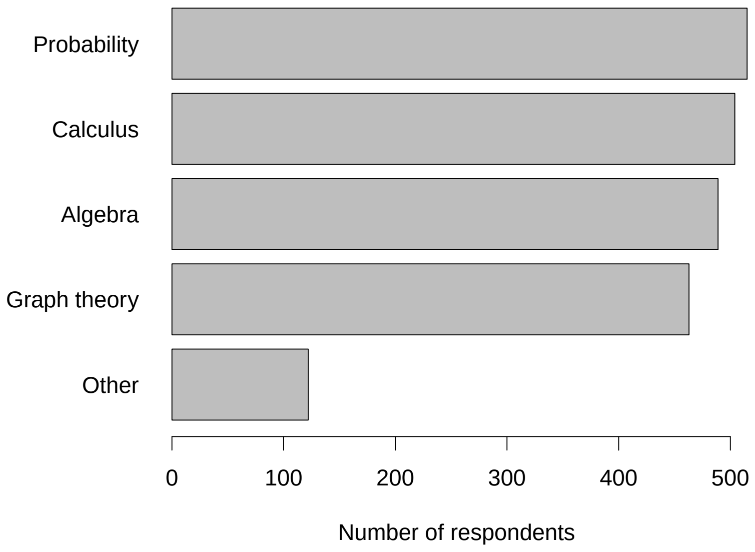

Figure S5: Number of respondents lacking knowledge in one of the key sub-disciplines of mathematics for ecology.
